# Mesenchymal stem cells generate distinct functional hybrids *in vitro* via cell fusion or entosis

**DOI:** 10.1038/srep36863

**Published:** 2016-11-09

**Authors:** Francesco Sottile, Francesco Aulicino, Ilda Theka, Maria Pia Cosma

**Affiliations:** 1 Centre for Genomic Regulation (CRG), The Barcelona Institute of Science and Technology, Dr. Aiguader 88, 08003 Barcelona, Spain; 2 Universitat Pompeu Fabra (UPF), Dr Aiguader 88, 08003 Barcelona, Spain; 3 ICREA, Pg. Lluís Companys 23, 08010 Barcelona, Spain

## Abstract

Homotypic and heterotypic cell-to-cell fusion are key processes during development and tissue regeneration. Nevertheless, aberrant cell fusion can contribute to tumour initiation and metastasis. Additionally, a form of cell-in-cell structure called entosis has been observed in several human tumours. Here we investigate cell-to-cell interaction between mouse mesenchymal stem cells (MSCs) and embryonic stem cells (ESCs). MSCs represent an important source of adult stem cells since they have great potential for regenerative medicine, even though they are also involved in cancer progression. We report that MSCs can either fuse forming heterokaryons, or be invaded by ESCs through entosis. While entosis-derived hybrids never share their genomes and induce degradation of the target cell, fusion-derived hybrids can convert into synkaryons. Importantly we show that hetero-to-synkaryon transition occurs through cell division and not by nuclear membrane fusion. Additionally, we also observe that the ROCK-actin/myosin pathway is required for both fusion and entosis in ESCs but only for entosis in MSCs. Overall, we show that MSCs can undergo fusion or entosis in culture by generating distinct functional cellular entities. These two processes are profoundly different and their outcomes should be considered given the beneficial or possible detrimental effects of MSC-based therapeutic applications.

Cell-to-cell fusion is a highly regulated key process involved in development and tissue homeostasis^1^,^2^. In particular cell fusion is required for fertilization, macrophage-derived giant cells and skeletal muscle formation, bone development and syncytiotrophoblast generation. As an example, trophoblast cells have a remarkable fusion capability that allows the formation of the syncytiotrophoblast, which is indispensable for the blastocyst implantation^3^. Importantly in injured tissues bone marrow derived cells (BMDC) can fuse *in vivo* with differentiated cells and form hybrids with regenerative potential^2^. In fact, bone marrow-derived hybrids were found in many organs such as brain, retina, liver, muscle and gut where they participated in the reestablishment of tissue function^4^,^5^,^6^,^7^,^8^,^9^,^10^,^11^,^12^. Based on these premises, several cell therapy approaches using BM-transplantation have been carried out to regenerate different tissues^13^,^14^,^15^,^16^,^17^,^18^,^19^.

On the other hand, heterotypic cell fusion has also been associated to cancer development and metastasis formation. In particular, cancer cells can fuse with different cell types, including stromal, epithelial and endothelial cells generating genetically instable hybrids^20^,^21^,^22^. Additionally, it was shown that macrophages or bone marrow-derived cells behave as fusion partners in several types of tumours^23^,^24^,^25^,^26^,^27^.

Cell fusion is also an essential approach to study somatic cell reprogramming mechanisms^28^,^29^,^30^,^31^,^32^. Indeed, it has been extensively used *in vitro* to investigate the activity of several transcription factors and pathways for their role in the enhancement of the reprogramming process^33^,^34^,^35^.

Taking into account all these previous reports, despite cells can spontaneously fuse both *in vitro* and *in vivo* with low efficiency^36^,^37^,^38^,^39^, cell-to-cell fusion is a critical biological process, which warrants investigation.

Recent studies have reported and characterized another form of cell-cell interaction, named entosis, which has been found in a variety of human tumours and can either play a pro-tumorigenic or a tumour suppressor role^40^. Entosis is a form of cell-in-cell structure originated by the active invasion of one living cell into another. It is caused by the loss of cell-matrix adhesion and it is mediated by adherent junctions and by the activity of the Rho-ROCK-actin/myosin pathways^41^,^42^,^43^,^44^,^45^,^46^.

Here we found that mesenchymal stem cells (MSCs) can either fuse, thereby forming heterokaryons, or be invaded by mouse embryonic stem cells (mESCs) through entosis. Moreover, we found that the ROCK-actin/myosin pathway is necessary for both mESC fusion and entosis but only for entosis in the case of MSCs. Importantly we showed that, contrary to cytoplasmic membrane fusion, nuclear membranes appear not to fuse directly. Instead cell division, disassociation and reassembly of the nuclear envelope allow the mixing and redistribution of parental chromosomes to the daughter cells, therefore generating synkaryons. Finally, considering the importance of MSC-based therapeutic applications, we implemented a straightforward method to purify either entotic or fusion–derived hybrids. In the future our approach and observations could be extended to investigate the outcome of these two profoundly different processes *in vivo*.

## Results

### MSCs form fusion and entosis-derived hybrids with ESCs in culture

**Figure 1:**
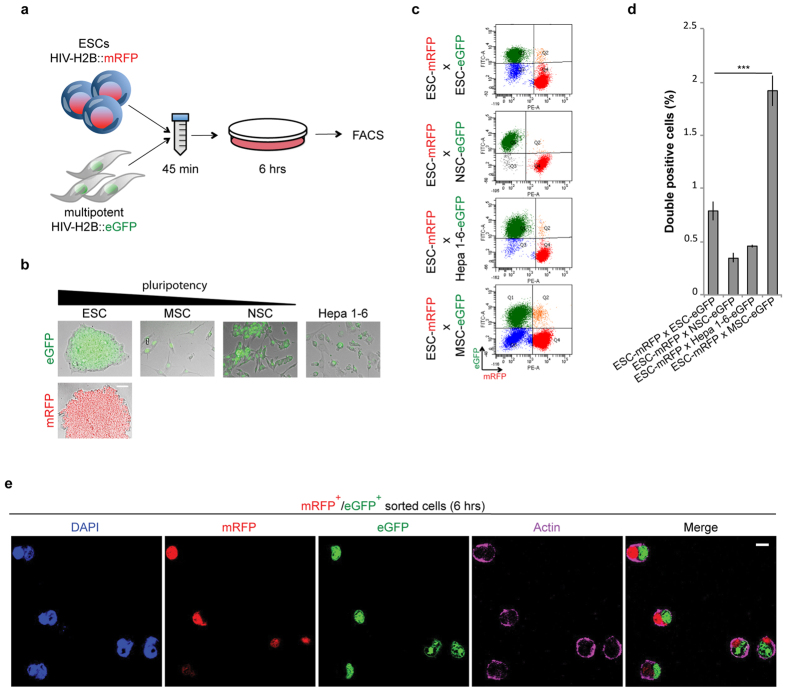
Mixed MSCs and ESCs form heterotypic hybrids *in vitro*. (**a**) Experimental scheme representing the co-culture condition to identify the best fusogenic cell lines. (**b**) Representative fluorescence micrograph of different cell types transduced with human immunodeficiency lentiviral particles carrying HIV-H2B::mRFP or eGFP (scale bar 50 μm). (**c**,**d**) Representative FACS analysis and quantification of eGFP^+^/mRFP^+^ cells derived from the indicated co-cultured cells. Data are represented as means ± SE (number of independent experiments n = 5) and statistical significance is represented by unpaired t-Test ***P < 0,001. (**e**) Confocal images of FACS sorted eGFP^+^/mRFP^+^ cells derived from ESC-mRFP and MSC-eGFP co-cultured after being 45 min in suspension and 6 hrs in adhesion (scale bar 10 μm).

In order to investigate the mechanism of cell-to-cell fusion process we set up an *in vitro* system to identify cell lines that fuse more efficiently in culture. To this purpose a panel of either somatic, multipotent or pluripotent murine cell lines with a reported fusion capability^33^,^47^,^48^,^49^,^50^ were modified to constitutively express H2B tagged with either enhanced green fluorescent protein (H2B-eGFP) or monomeric red fluorescent protein (H2B-mRFP) (Fig. 1a,b and Supplementary Figure S1a). ESCs-mRFP were mixed in suspension for 45 min with either ESCs-eGFP, MSCs-eGFP, neural stem cells (NSCs)-eGFP or with hepatocarcinoma cells (Hepa-1–6)-eGFP, then cultured for 6 hrs and finally analysed by flow cytometry (Fig. 1c). When ESCs were mixed together with NSCs or with Hepa 1–6 or with themselves, we detected very few hybrids (eGFP^+^/mRFP^+^ cells) (Fig. 1d). Conversely, we identified almost 2% of double positive cells when ESCs were co-cultured with MSCs (Fig. 1d). Furthermore, confocal microscopy analysis on FACS-sorted eGFP^+^/mRFP^+^ cells confirmed the presence of two nuclei into a unique cytoplasm (Fig. 1e). Finally, cell cycle analysis performed by DAPI staining showed, as expected for hybrid cells, higher DNA amount as compared to the parental cell types (Supplementary Figure S1b). These data indicate that MSCs and ESCs spontaneously form hybrid cells already after 6 hrs of co-culture and with a higher efficiency compared to the other tested cell lines.

**Figure 2:**
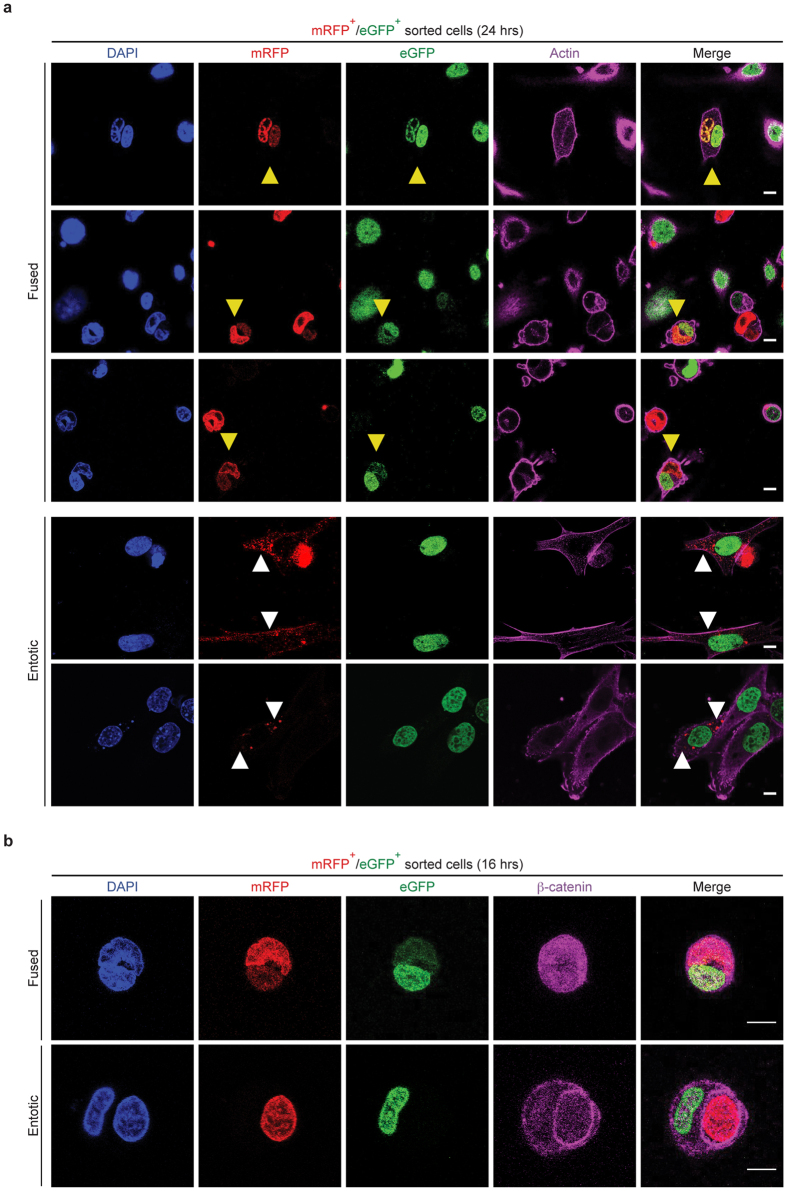
ESCs spontaneously fuse with MSCs or undergo entosis. (**a**) Representative confocal micrograph of FACS-sorted eGFP^+^/mRFP^+^ cells 24 hrs after the co-culture of ESCs-mRFP and MSCs-eGFP. Internalised and degraded ESCs-mRFP within MSCs-eGFP and heterokaryon cells are indicated by white and yellow arrowheads, respectively (scale bar 10 μm). (**b**) FACS-sorted eGFP^+^/mRFP^+^ cells were immunostained for ß-catenin to distinguish fused (upper panel) versus entotic (lower panel) cells (scale bar 10 μm).

To better examine the nature of MSC/ESC-derived hybrids, we FACS-sorted the eGFP^+^/mRFP^+^ cells after 6 hrs of co-culture and the resulting hybrids were analysed by confocal microscopy after 24 hrs. Surprisingly we noticed two distinct phenotypes. First, as expected, the heterokaryons were characterised by the presence of two nuclei derived from the parental cells into the same cytoplasm where the eGFP-positive nucleus incorporated H2B-mRFP proteins from the fusion partner and *vice versa* (Fig. 2a, yellow arrowheads). In addition, beside fusion-derived heterokaryons, we observed MSC-like cells with punctate mRFP signal into the cytoplasm. These cells displayed a phenotype similar to previously reported entotic process that, to date has not been described in MSCs (Fig. 2a, white arrowheads).

Entosis occurs when a living cell invade another cell’s cytoplasm caused by the detachment from the extracellular matrix (ECM)^41^,^42^,^43^,^44^. To further investigate the two observed phenotypes, we analysed eGFP^+^/mRFP^+^-sorted cells by time-lapse microscopy. Interestingly, we noticed the presence of both cell fusion-derived hybrids and entosis-derived hybrids. Cell fusion-derived hybrids (heterokaryons) were characterized by the presence of 2 nuclei sharing the same cytoplasm that were capable to exchange H2B-eGFP or H2B-mRFP (Supplementary Movies S1 and S2). In contrast, in entosis-derived hybrids, the ESCs appeared to be inside a large vacuole, internalized into MSCs and histone exchange was never detected (Supplementary Movies S3 and S4). Remarkably, we noticed that MSC-like cells with punctate cytoplasmic mRFP resulted from the entotic process (Supplementary Movie S5), similarly to previous observations^51^,^52^. It has been reported that internalised cells after co-culture of mammary epithelial cells are initially alive and can eventually divide, be killed or released^41^. Similarly, we found that entosis of ESCs into MSCs could recapitulate these phenotypes (Supplementary Movies S6–S8).

To further confirm these observations, eGFP^+^/mRFP^+^ cells were analysed at earlier time point (16 hrs after sorting) by immunostaining against β-catenin to highlight the plasma membrane. Analysis by confocal microscopy demonstrated either the complete internalization of ESCs within MSCs or the fusion between the two different cell types generating thereby entosis-derived hybrids or fusion-derived hybrids (Fig. 2b). Moreover, this result was also confirmed by transmission electron microscopy (TEM) ultra-micrograph (Supplementary Figure S2a).

We performed a number of control experiments to exclude that MSC-like cells, which included punctate mRFP signal into the cytoplasm, could derive from a phagocytic response due to the differentiation of MSCs into macrophage-like cells in our co-culture condition. First, neither ESCs nor MSCs expressed the macrophage cell marker Mac-1 suggesting that none of these cell types differentiate into phagocytic cells in our culture conditions (Supplementary Figure S2b). Secondly, since cell opsonisation can enhances macrophage-mediated phagocytosis^53^, we co-cultured ESCs and MSCs after ESC opsonisation using an antibody against the ESC cellular marker SSEA-1. Even in this experimental condition we did not observe any increase in the amount of eGFP^+^/mRFP^+^ double positive cells confirming that MSCs did not differentiate into macrophage-like cells (Supplementary Figure S2c). Finally, MSCs and ESCs mixed together were examined for phosphatidylserine (PS) exposure with Annexin V protein. PS is an “eat-me” signal exposed on the outer leaflet of dying cell’s plasma membrane, which is recognised by phagocytic cells^54^. Annexin V^+^ cells were not detected after 6 hrs of co-culture suggesting that the PS pro-phagocytic signal is not involved in this process (Supplementary Figure S2d). Therefore, these data strongly suggest that phagocytic clearance does not explain the presence of punctate mRFP signal into the cytoplasm of MSC-like cell.

Overall, here we show that MSCs and ESCs can spontaneously form either heterokaryons or undergo entosis when cultured together.

### Fused and entotic cells can be distinguished by surface markers

**Figure 3:**
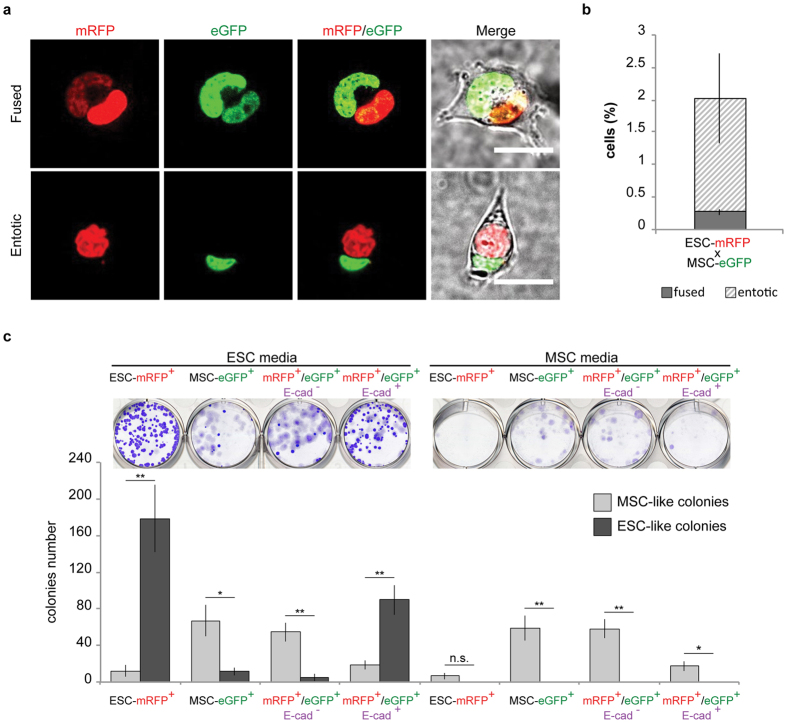
Fused or entotic cells can be distinguished and quantified by E-cadherin staining. (**a**) Confocal images of living FACS-sorted fused and entotic cells (scale bar 10 μm). (**b**) Quantification of fused versus entotic cells. Co-cultured cells were stained for E-cad to quantify fused (eGFP^+^/mRFP^+^/E-cad^+^) and entotic (eGFP^+^/mRFP^+^/E-cad^−^) cells. Data are represented as means ± SE (number of independent experiments n = 15). (**c**) Colony forming unit assay (CFU-F) of parental mESCs-mRFP, MSCs-eGFP, eGFP^+^/mRFP^+^/E-cad^−^ cells and eGFP^+^/mRFP^+^/E-cad^+^ cells 6 days after sorting. Representative images of crystal violet stained colonies (upper images) and quantitative analysis of colony forming rate of mESCs-mRFP, MSCs-eGFP, eGFP^+^/mRFP^+^/E-cad^−^ entosis-derived hybrids and eGFP^+^/mRFP^+^/E-cad^+^ fusion-derived hybrids (lower plots). Data are represented as means ± SE (number of independent experiments n = 4) and statistical significance is represented by unpaired t-Test *P < 0,05, **P < 0,01.

Since cell fusion and entosis give rise to functionally distinct hybrids, it is important to discriminate heterokaryons from entotic cells and study them separately. When MSCs and ESCs fuse together, the resulting hybrids are characterized by a mixed plasma membrane. On the contrary membrane/cytoplasm mixing does not occur in case of entosis. Therefore we reasoned that it is possible to discriminate fused from entotic cells using cell specific plasma membrane markers. In particular, ESCs express epithelial cadherin (E-cad) at the plasma membrane that, in contrast, it is not expressed in the MSCs (Supplementary Figure S3a,b). Given this specific feature, it is therefore possible to distinguish and isolate by FACS either fused (eGFP^+^/mRFP^+^/E-cad^+^) or entotic (eGFP^+^/mRFP^+^/E-cad^−^) cells based on the colour pattern (Fig. 3a and Supplementary Figure S3c). After 6 hrs of co-culture, approximately 0,25% of cells were eGFP^+^/mRFP^+^/E-cad^+^ and 1,75% were eGFP^+^/mRFP^+^/E-cad^−^ (Fig. 3b), demonstrating that it is possible to quantify and enrich both entosis and fusion-derived hybrids after co-culture. To further confirm the efficacy of our E-cad-based purification system we analysed eGFP^+^/mRFP^+^ by imaging flow cytometry^55^. This method allowed us to simultaneously collect real-time images of each event in the flow stream during the FACS analysis. We observed that the heterokaryons were decorated by the E-cad ring on plasma membrane, while the entotic derived hybrids were negative for E-cad immunostaining (Supplementary Figure S3d). Of note, out of the fused cells we never observed synkaryons immediately after FACS-sorting.

Finally, to assess whether cell fusion and entosis-derived hybrids could generate ESC-like and MSC-like colonies from single cells we performed a colony forming unit assay (CFU-F). We FACS-sorted entosis- (eGFP^+^/mRFP^+^/E-cad^−^), fusion-derived hybrids (eGFP^+^/mRFP^+^/E-cad^+^) after 6 hrs of co-culture and both ESCs-mRFP and MSCs-eGFP as controls. The different cell types were plated either in ESC or MSC media. We observed that MSCs-eGFP grew from single cells and formed colonies in both culturing media while ESCs-mRFP did not survive in MSC medium (Fig. 3c). In ESC-permissive growth conditions, both fusion and entosis-derived hybrids generated colonies from single cells. Importantly, fusion-derived hybrids (RFP^+^GFP^+^Ecad^+^ cells) formed ESC-like colonies, although in a reduced number if compared to the control ESCs-mRFP cells. In contrast, in MSC media, fusion-derived hybrids (RFP^+^GFP^+^Ecad^+^ cells), which retain ESC-like feature, did not survive while entosis-derived hybrids (RFP^+^GFP^+^Ecad^−^ cells) formed MSC-like colonies with a growth rate comparable to the MSC control (Fig. 3c). Thus, heterokaryons as well as entosis-derived hybrids can proliferate *in vitro* after sorting and generate ESC- and MSC-like colonies respectively.

These data show that we developed a simple purification approach that allows the enrichment of either entotic or fusion-derived hybrids, which can thereby be studied separately.

### Cytoskeleton components are essential for fusion and entosis

**Figure 4:**
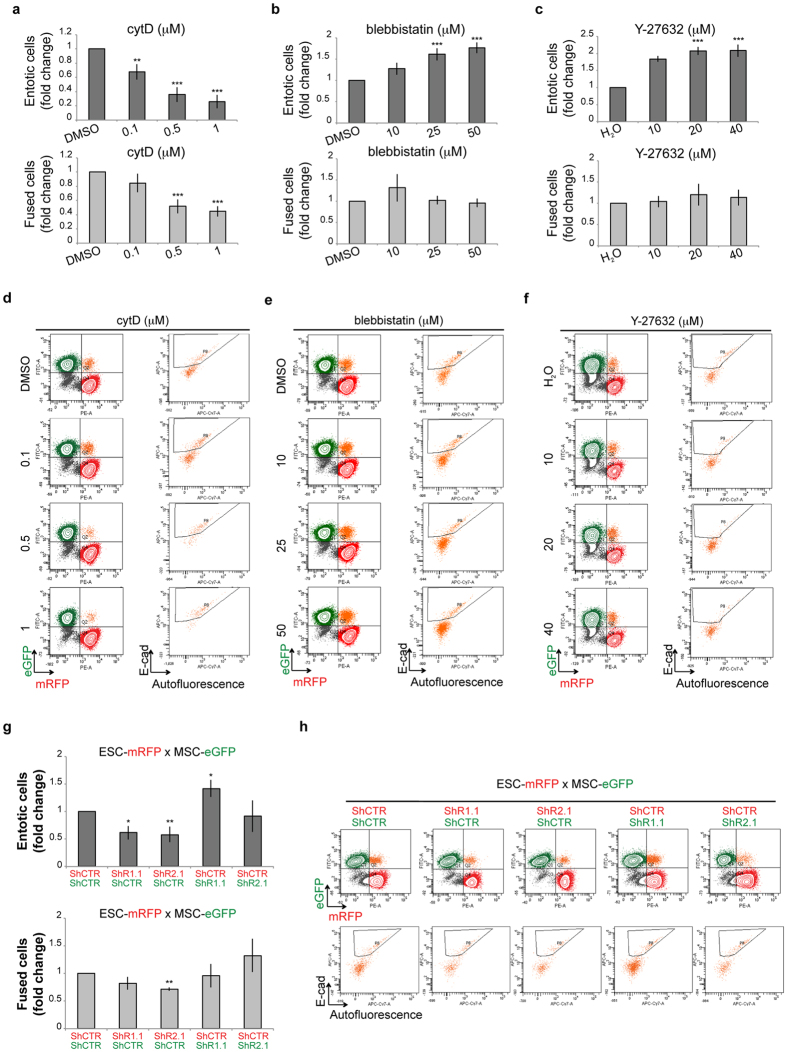
Cytoskeleton components are essential for fusion and entosis. (**a**–**c**) Quantification of fused and entotic cells treated with an increasing concentration of cytochalasinD (cytD) (**a**) blebbistatin (**b**) and rock inhibitor Y-27632 (**c**). Data are represented as means ± SE (number of independent experiments n = 9) and statistical significance is represented by unpaired t-Test *P < 0,05, **P < 0,01, ***P < 0,001. (**d**–**f**) Representative FACS analysis of fused and entotic cells treated with an increasing concentration of cytochalasin D (cytD) (**d**) blebbistatin (**e**) and rock inhibitor Y-27632 (**f**). (**g,h**) Quantification and representative FACS analysis of fused and entotic cells after co-culture of ESCs and MSCs infected with lentiviruses carrying *shCTR, shR1.1* or *shR2.1*. Data are represented as means ± SE (number of independent experiments n = 5) and statistical significance is represented by unpaired t-Test *P < 0,05, **P < 0,01, ***P < 0,001

Entosis requires active actin polymerization, myosin II contraction and the activity of the Rho signalling pathway^41^,^42^,^43^,^44^,^45^,^46^. To investigate whether cell fusion requires the same machinery, mixed cells were treated either with cytochalasin D (cytD), an inhibitor of actin polymerization, or with the myosin II inhibitor blebbistatin. Inhibition of actin polymerization suppressed both entosis and fusion in a dosage dependent manner suggesting an active role for actin in both processes (Fig. 4a,d). Myosin II contraction inhibition has been shown to suppress entosis in epithelial breast cancer cells^41^. Surprisingly, mixed MSCs and ESCs treated with blebbistatin showed higher entotic capability when compared to the control but did not affect cell fusion (Fig. 4b,e). These results suggest that the cytoskeleton plays different functions in both processes in the two different cell types.

To further investigate this phenotype we decided to examine Rho GTPases activity. In particular we analysed the role of Rho GTPases downstream effectors, the Rock proteins, which are both actin and myosin II regulators. We therefore assessed whether Rho signalling plays a role in either entosis or fusion of co-cultured MSCs/ESCs by using the Y-27632 inhibitor^56^. Similar to blebbistatin, Y-26732-treated cells exhibited an increased entotic capability whereas cell fusion was not affected (Fig. 4c,f). Of note, cytD, blebbistatin and Y-27632 treatment altered morphology of MSCs and ESCs, indicating that these drugs affected the cytoskeleton (Supplementary Figure S4a–c). Altogether, these data indicate that actin polymerization is essential for entosis as well as for fusion of ESCs with MSCs, whereas myosin II contraction is important only for cell internalization, i.e. for entosis, differently from what previously reported for epithelial breast cancer cells.

To further strengthen these observations, MSCs and ESCs were transduced with lentiviral vectors expressing small hairpin RNAs (shRNA) to knock-down either *Rock1* or *Rock2* (Supplementary Figure S4d). Based on the silencing efficiency we selected one shRNA per protein, *shR1.1* and *shR2.1* (Supplementary Figure S4d,e). Interestingly, *Rock1* and *Rock2* downregulation in ESCs resulted into an inhibitory effect of entosis (Fig. 4g,h). Silencing in ESCs of *Rock2* inhibited cell fusion, which showed a tendency to decrease also upon *Rock1* downregulation (Fig. 4g,h). This effect was similar to what observed with the cytD treatment (Fig. 4a). Instead, in MSCs only *Rock1* silencing enhanced entosis, but not cell fusion (Fig. 4g,h), similar to blebbistatin and Y-27632 treatments (Fig. 4b,c). These data indicate that the Rho-Rock-actin/myosin pathway plays different roles in ESCs and MSCs. While the Rho pathway is specifically required in ESCs for both fusion and entosis processes, the downregulation of *Rock1* in MSCs turned them into a more permissive partner for entosis but not for cell fusion.

To rule out the possibility that cytoskeleton perturbations could enhance cell doublets formation and that these aggregates could have been erroneously recognised as hybrids, we performed a cell-aggregate analysis based on a stringent gating strategy. In particular, we analysed the total amount of cell doublets after cytD, blebbistatin and Y-27632 treatment (Supplementary Figure S5a) and in *Rock1/2* KD co-cultures (Supplementary Figure S5b). Importantly, no significant variation in the percentage of total cell doublets was observed in all the experimental conditions (Supplementary Figure S5c,d) indicating that cell doublet formation is not altered by drugs treatment or by the *Rock1/2* silencing. Additionally, these observations are consistent with previous studies showing that Rock and myosin-based contraction of the actin cytoskeleton are required for cell-cell adhesion and maintenance of sarcoma cell doublets^57^,^58^. Overall, these data indicate that cytoskeleton rearrangement and the Rho-Rock pathway are important for both entosis and cell fusion. In particular, the Rho-Rock-actin/myosin pathway is specifically required in ESCs for both fusion and entosis processes, while this pathway turned MSCs into a more permissive partner for entosis but not for cell fusion.

### Hetero to synkaryon transition requires cell division

Heterokaryons can convert into synkaryons during reprogramming of somatic cells *in vivo* and this might represent an important process for tissue regeneration. On the other hand, the possible aberrant chromosome segregation during hetero to synkaryon transition could ultimately result in the formation of hybrid cells characterized by genomic alterations. It is therefore important to study how synkaryons are generated. In addition, whether the transition from hetero to synkaryons in mammalian cells involves nuclear membrane fusion of the two parental nuclei or a different mechanism is still unclear.

**Figure 5:**
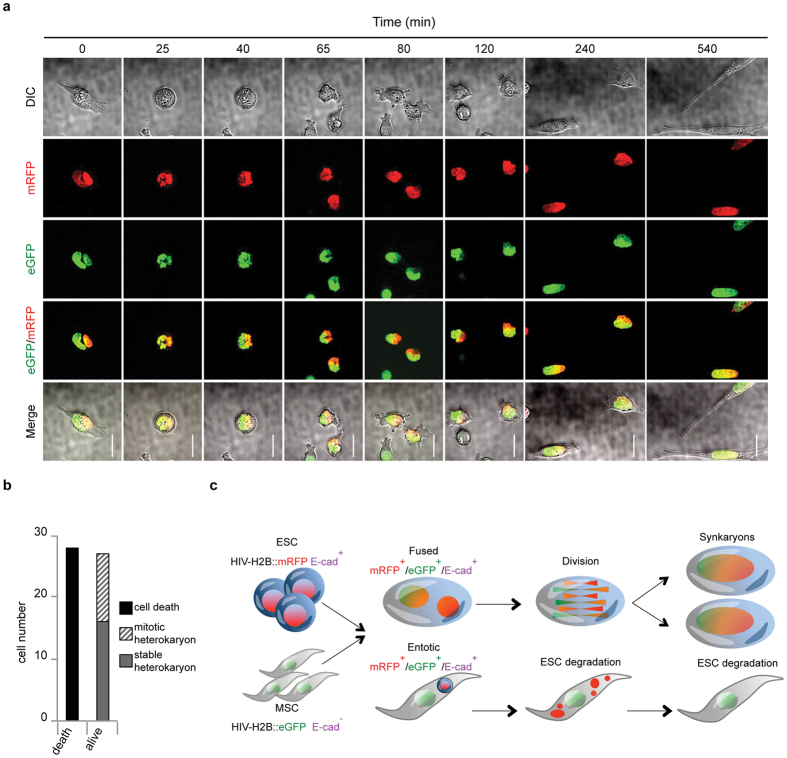
Hetero to synkaryon transition is mediated by mitosis. (**a**) Snapshots of the time-lapse of ESCs and MSCs derived heterokaryon referred to movie S11 (scale bar 10 μm). FACS-sorted heterokaryons were plated and after 2 hrs the attached cells were processed for time-lapse imaging for additional 12 hrs. (**b**) Quantification of heterokaryon cells fate over approximately 12 hrs after sorting. Data represent the total number of heterokaryon analysed (heterokaryons analysed n = 55). (**c**) Schematic representation of the optimised experimental protocol to study heterokaryon or entotic cell fate.

To examine how the two distinct nuclei of MSCs and ESCs fuse to form synkaryon starting from living heterokaryons, MSCs-eGFP/ESCs-mRFP derived hybrids were FACS-sorted as described above (eGFP^+^/mRFP^+^/E-cad^+^) and analysed by time-lapse microscopy. As expected, the majority of the heterokaryons died quickly after sorting probably due to genomic instability as quantified in Fig. 5b (Supplementary Movie S9). Interestingly, among the surviving fusion-derived hybrids, the heterokaryons that underwent cell division mixed the two genomes to generate two-daughter synkaryon cells (Fig. 5a,b and Supplementary Movies S10 and S11). These data provide quantitative evidence that transition from hetero to synkaryons in MSC/ESC-derived hybrids occurs through cell division rather than from nuclear membrane fusion.

## Discussion

Cell-to-cell fusion is an essential process for development and tissue repair^1^,^2^. Beside these positive effects, aberrant segregation of chromosomes in the hybrid cells could result into cancer generation^23^. Entosis, a cell-in-cell invasion, has been firstly characterized in breast cancer cells^41^, and likewise cell fusion it was also recently seen to play a role during early development. Indeed, trophoblast cells not only fuse into a syncytia placental tissue but recent studies demonstrated that trophoblast cells can also remove uterine luminal epithelial cells through entosis, facilitating embryo implantation^59^.

In this report, we showed that MSCs and mESCs can spontaneously form either entotic or fusion-derived hybrids in co-culture, although with low efficiency. We developed a simple purification protocol based on surface markers that allowed the quantification and purification of both entotic and heterokaryon cells (Fig. 5c). Heterokaryons and entosis-derived hybrids can proliferate *in vitro* and generate ESC- and MSC-like colonies respectively. It will be interesting to analyse their ability to differentiate toward different lineages, although currently this is technically challenging due to the low number of hybrids obtained from sorting.

Notably, it has been demonstrated that different cell types, including MSCs, neuronal cells, endothelial cells and others, can exchange cytosolic elements such as vesicles and mitochondria through tunneling nanotubes (TNTs)^60^,^61^,^62^,^63^,^64^. Although we did not directly investigate the occurrence of TNTs, we cannot exclude this possibility in a limited number of cases.

Cytoskeleton rearrangement and the Rho-ROCK pathway are critical for both MSC differentiation and ESC self-renewal^65^,^66^. Here we showed that the Rho-Rock-actin/myosin pathway is important for both cell fusion and entosis in ESCs. On the contrary, its inhibition turned the MSCs into a more permissive partner for entosis but not for cell-fusion. These observations lead us to speculate that a reduction of MSC cortex tension might provoke the formation of a more fluid plasma membrane that facilitates MSC deformation and finally ESC invasion.

In this report we also aimed to study how synkaryons are generated. In yeast, during mating, the two haploid nuclei fuse their nuclear membrane to generate a diploid nucleus^67^. Similarly, in sea urchin fertilization sperm and egg pronuclear membrane fuse to mix the genomic material^68^. However, during mammalian fertilization sperm-derived and egg-derived chromosomes condense at the first mitotic prophase and mix on the metaphase equator initiating the first mitotic division^69^. Surprisingly, in the case of cell fusion we found that transition from hetero- to synkaryons occurs through cell division rather than by nuclear membrane fusion, whereas in the case of entosis internalized cells can be released, undergo cell division or be degraded, confirming earlier experimental observations^41^. We therefore demonstrated that heterokaryon cells are transient precursors that are indispensable to generate *bona fide* synkaryon daughter cells through mitosis-mediated mechanism. On the other hand, whether the efficiency of this process can be cell-type specific will be a matter of future investigation.

MSCs are an important adult stem cell resource with great potential for regenerative medicine. Therefore it is essential to fully investigate either the beneficial or detrimental effects of heterokaryons or entosis-derived hybrids in MSC-based therapeutic applications. Future studies will be needed to investigate how synkaryons divide and segregate their chromosomes to generate either stable or instable hybrids with regenerative or tumorigenic potential, respectively. Moreover it will be interesting to characterize whether the entotic activity of MSCs could contribute to either tumour specific suppression or progression.

## Material and Methods

### Co-cultures

At day 1 ESCs, NSCs, Hepa 1–6 and MSCs were plated respectively at 4*10^3^ cell/cm^2^, 15*10^3^ cell/cm^2^, 10*10^3^ cell/cm^2^ and 10*10^3^ cell/cm^2^. At day 3 each cell type was detached with cell dissociation buffer (GIBCO 13151–014) mixed in ESC medium in a 15 ml falcon tube, pelleted by centrifugation at 300 rcf for 5 min and the supernatant was aspirated. The pellet was re-suspended in two volumes of ESC medium, incubated at 37 °C for 45 min and plated in gelatine-coated dishes for other 6 hrs.

### Time-lapse imaging

FACS-sorted cells were plate into Time-lapse gelatin (Millipore ES-006-B)-coated Thermo Scientific Nunc Lab-Tek chambered coverglass and images were acquired on Andor Revolution XD inverted microscope (Olympus). All imaging of living cells was performed in incubator chamber at 37 °C and 5% CO_2_.

## Additional Information

**How to cite this article**: Sottile, F. *et al.* Mesenchymal stem cells generate distinct functional hybrids *in vitro* via cell fusion or entosis. *Sci. Rep.* **6**, 36863; doi: 10.1038/srep36863 (2016).

**Publisher’s note:** Springer Nature remains neutral with regard to jurisdictional claims in published maps and institutional affiliations.

## Supplementary Material

Supplementary Information

Supplementary Information

Supplementary Information

Supplementary Information

Supplementary Information

Supplementary Information

Supplementary Information

Supplementary Information

Supplementary Information

Supplementary Information

Supplementary Information

Supplementary Information
