# Supplementary material for: Mesenchymal stem cells generate distinct functional hybrids in vitro via cell fusion or entosis
Source: Sci Rep. 2016 Nov 9;6:36863. doi: 10.1038/srep36863 (PMC5101832; doi:10.1038/srep36863)
Supplement: Supplementary Information [file srep36863-s12.pdf]

Inventory of Supplementary Information

Mesenchymal stem cells generate distinct functional hybrids *in vitro* via cell fusion or entosis

Francesco Sottile, Francesco Aulicino, Ilda Theka and Maria Pia Cosma

1. Supplementary Material and Methods
2. Supplementary Reference
3. Supplementary Figures Legends
4. Supplementary Movies Legends
5. Supplementary Figures

1. Supplementary Material and Methods

Cell lines

GS-1 ESCs (Sv/129) were maintained in gelatin (Millipore ES-006-B)-coated plates with DMEM supplemented with 15% foetal bovine serum (FBS), L– glutamine (2mM) (Thermo Fisher Scientific 25030024), penicillin (100U/ml), streptomycin (100µg/ ml) (15140122 Thermo Fisher Scientific), sodium pyruvate (1mM) (11360039 Thermo Fisher Scientific), non-essential amino acid (NEAA) (0,1mM) (11140035 Thermo Fisher Scientific), 2-mercaptoethanol (0,5mM) (31350010 Thermo Fisher Scientific) and ESGRO mLif (1000U/ml) (ESG1107 Millipore).

GIBCO MSCs (C57BL/6) (S1502-100) were maintained in DMEM/F-12-GlutaMAX supplemented with 10% fetal bovine serum (FBS), penicillin (100U/ml), streptomycin (100µg/ ml).

Hepa1-6 (C57L) were purchased from ATCC and maintained in culture with DMEM supplemented with 10% fetal bovine serum (FBS), penicillin (100U/ml), streptomycin (100µg/ ml).

NS-GiP cells carrying the regulatory sequences of the mouse *Oct4* gene driving GFP expression and puromycin resistance were a gift from Dr. A. Smith (Cambridge Stem Cell Institute). NS-GiP cells were obtained from HP165 mice and maintained in Poly-l-Ornithine (SIGMA P4957) and Laminin (SIGMA L2020) coated plate and cultured in RHB-A supplemented with penicillin (100U/ml), streptomycin (100µg/ ml), bFGF (Peprotech AF-100-18B) (10ng/ml) and EGF (Peprotech AF-100-15C) (50ng/ml) as previously described[1, 2].

Flow cytometry

After 6 hours of co-culture, cells were detached with cell dissociation buffer (GIBCO 13151-014) pelleted by centrifugation at 300 rcf for 5 min, the pellet was washed once and resuspended in PBS with 5% FBS plus DAPI (SIGMA 09542). The percentage of $eGFP^+$ and $mRFP^+$ cells was quantified using a BD LSR Fortessa and analysed by BD FACSDiva. Uninfected cells were used as negative control. The less abundant single cell population either ESC-mRFP or MSC-eGFP, in each flow cytometry experiment was considered as the limiting factor for hybrids formation. The percentage of hybrids ($eGFP^+/mRFP^+$) was normalized with respect to the less abundant single positive population. The samples were filtered with 35µm mesh size

filters (Corning Life Sciences 352235) before FACS analysis to avoid aggregates.

For the analysis and/or for sorting of entotic and heterokaryon cells the sample were prepared as previously described and harvested cells were incubated with antibody against E-cad (0,5 μg/10^6 cells, Biolegend 147308) in PBS with 5% FBS with DAPI for 20 min at 4°C, washed once in PBS plus 5% FBS, FACS sorted or analysed with BD Influx and BD LSR Fortessa respectively. Cells stained with Rat IgG1 (0,5 μg/10^6 cells, Invitrogen R104) were used as negative control.

For imaging flow cytometry all samples were analysed using Amnis imaging flow cytometer that simultaneously collects multi-mode images of each event in the flow stream including brightfield, darkfield and up to twelve fluorescence colours. The 60x magnification objective was used to provide a field of view of 40 x 170 μm. In each experiment between $6 \cdot 10^4$ to $8 \cdot 10^5$ events were recorded to ensure a sufficient number of events for the analysis. Images were analysed by the Cytometry and cell sorting facility at Institut d'investigacions Biomèdiques August Pi i Sunyer (IDIBAPS) using IDEAS software, unstained or single stained cells were used as compensation controls to generate compensation matrices (http://www.idibaps.org/core-facilities/7/cytometry-and-cell-sorting-facility).

Macrophage marker Mac-1 was detected by flow cytometry using α-Mac-1 antibody (0,5 μg/10^6 cells, eBioscience 17-0112) in PBS with 5% FBS plus DAPI for 20 min at 4°C, washed once in PBS plus 5% FBS and analysed with BD LSR Fortessa. Cells stained with Rat IgG2, k (0,5 μg/10^6 cells, eBioscience 17-4321) were used as negative control. The samples were filtered with 35μm mesh size filters before FACS analysis to avoid aggregates.

Phosphatidylserine exposure was detected by flow cytometry using APC Annexin V (BD Pharmingen 550474) following the manufacturer's instructions and analysed using BD LSR Fortessa. Unstained cells were used as negative control. The samples were filtered with 35μm mesh size filters before FACS analysis to avoid aggregates.

Antibody against SSEA-1 was used to opsonize ESCs (0,5μg/10^6 cells) (Biolegend 125607) in ESC media for 20 min at 4°C, washed once and mixed with MSCs as describe above. Cells stained with Mouse IgM, k (0,5 μg/10^6 cells, BD Pharmingen 553409) were used as negative control. The samples were filtered with 35μm mesh size filter before FACS analysis to avoid aggregates

Immunofluorescence staining

Cells were FACS-sorted directly on microscope glass slide for 6 hrs analysis or plated into gelatin-coated Thermo Scientific Nunc Lab-Tek chambered coverglass (155411) for 16 hrs and 24 hrs analysis. Cells were fixed with 4% paraformaldehyde for 15 min at room temperature, and then washed twice with PBS. Fixed cells were permeabilised in PBS-Triton X-100 (0,1%) (SIGMA T8787) and then incubated in blocking solution containing 4% BSA (SIGMA A7906) and 0.1% triton X-100 for 1 h at room temperature. The cells were then incubate overnight at 4 °C in blocking solution containing the primary antibody. The next day, the cells were washed three times with PBS-Triton X 100 (0,1%) and then incubated with the secondary antibody for 1h at room temperature. The primary antibodies used are listed in Supplementary Table S1. Goat anti-mouse IgG, (1:1000, Life Technologies A21050) conjugated to Alexa Fluor-633 were used as secondary antibodies and as negative control.
Nuclear staining was performed with DAPI (SIGMA 09542).

Transmission Electron Microscopy (TEM)

Cells were fixed with 2.5% glutaraldehyde in phosphate buffer 0,1M (PB) at room temperature for 1 hr and washed two times with cold 0,1M PB. Fixed cells were then processed for TEM and analysed by the electron cryo-microscopy unit at Centres Científics i Tecnològics de la Universitat de Barcelona (CCiTUB) http://www.ccit.ub.edu/EN/tb04.html.

Cell Cycle

Cells were detached with 0.05% Trypsin-EDTA (SIGMA 25300-054) and collected by centrifugation at 300 rcf for 5 min. The cell pellet was resuspended and fixed overnight in 3ml cold 70% ethanol. After fixation, the cells were centrifuged at 300rcf for 10 min at room temperature. The pellet was washed twice in 1ml PBS. During each wash, the cells were pelleted at 300rcf for 5 min at room temperature. Then cells were resuspended in DAPI solution (SIGMA 09542) (5µg/ml/10^6 cells in PBS) and incubated for 30 min on ice. Samples were analysed by BD LSR Fortessa and by BD FACSDiva.

Colony forming unit assay (CFU-F)

Entotic, heterokaryon and parental control cells were isolated as previously described and pleated for colony formation assay in 12-well plates at low density (500 cells/well). The medium was replaced every 2 days and 6 days after sorting cells were

fixed with 4% paraformaldehyde for 5 min at room temperature and then washed twice with PBS. Fixed cells were incubated 10 min at room temperature in methanol 100%, washed twice with PBS and then stained for 20 min at room temperature with crystal violet solution (0,5% crystal violet, 20% methanol in PBS). The stained cells were washed three times with deionized water and the number of colonies was counted.

Constructs

Lentiviral vector HIV-H2B::mRFP was purchased from Addgene (# 18982)[3]. The PCR product derived from pH2B-eGFP (Addgene #11680)[4] containing part of the H2B tagged with the eGFP was digested with EcoRI/ClaI and subcloned into the lentiviral vector HIV-H2B::mRFP.

Short hairpins targeting *Rock1* (*shRock1.1* and *shRock1.2*), *Rock2* (*shRock2.1* and *shRock2.2*) and a short hairpin control (*shCTR*) were cloned into the pLKO.1-Hygro lentiviral vector (Addgene plasmid #24150), following the manufacturer instructions (http://www.addgene.org/tools/protocols/plko/).

pCMV-ΔR8.2 dvpr (Addgene #8455) and pCMV-VSV-G (Addgene #8454) were used as lentiviral packaging constructs.

The oligonucleotides used to generate the HIV-H2B::eGFP and the short hairpins were purchased from SIGMA and listed in Supplementary Table S2.

All constructs used in this study will be made publicly available to the scientific community on Addgene (https://www.addgene.org/)

Virus preparation and infection

For infection, lentiviral particles were produced following the RNA interference Consortium (TRC) instructions for lentiviral particle production and infection in 6-well plates (http://www.broadinstitute.org/rnai/public/). At day 1 HEK293 cells were plated at $5*10^4$ cell/cm^2 in p150 plates. The day after plating, the cells were co-transfected with 30 µg pHIV-H2B::mRFP, HIV-H2B-eGFP, pLKO-*shRock1.1*, pLKO-*shRock1.2*, pLKO-*shRock2.1*, *pLKO-shRock2.2* or pLKO-*CTR* 19,5 µg pCMV-DR8.2, and 10,5 µg pCMV-VSV-G, using calcium phosphate transfection kit (Clontech 631312). ESCs, NSCs, Hepa1-6 and MSCs were plated at $5*10^4$ cell/cm^2 density onto gelatin-coated 6-well plates the day before transduction. The lentiviral-containing medium was harvested from HEK293T cells at 48, 72 and 96 h after

transfection, filtered, and ultracentrifuged with a Beckman SW28 rotor at 22000rpm for 2 hrs at 4°C. Concentrated lentiviral particles were resuspended in 100µl of PBS and 5µl added to ESCs, NSCs, Hepa1-6 and MSCs. Cells transduced with short hairpin RNA constructs were washed twice in PBS and hygromycin selection (50 µg/ml) was applied. Cells transduced with pHIV-H2B::mRFP, HIV-H2B::eGFP were washed twice in PBS and maintained in culture 5 more days and then FACS-sorted based on fluorescent intensity.

RNA extraction and quantitative PCR detection of mRNA

RNA was extracted and purified using RNAeasy kits (QIAGEN), according to the manufacturer instructions. Total RNA was treated with DNAse I (Qiagen) to prevent DNA contamination. The cDNA was produced with SuperScript II Reverse Transcriptase kits (Life Technologies) starting from 1 µg mRNA. Real-time quantitative PCR reactions from 8,3 ng of cDNA were set up in triplicate using a LightCycler SYBR Green I Master PCR machine (Roche 4887352001). For oligo sequences see Supplementary Table S2.

Western Blot

Cells were harvested and washed twice with PBS, after each wash cells were pelleted at 300 rcf for 5 min at room temperature. Cell lysis was performed on ice for 15 min, in RIPA buffer (SIGMA R0278) containing protease inhibitors (SIGMA P8340) and phosphatase inhibitors (SIGMA P5726). Insoluble material was pelleted by centrifugation at 16,000 rcf for 30 min at 4 °C. Protein concentrations were determined using the Bradford assay (BIO-RAD 500-0006). The extracts were mixed with 4x sample buffer (40% glycerol, 240 mM Tris/HCl, pH 6.8, 8% SDS, 0.04% bromophenol blue, 5% β-mercaptoethanol), denatured at 99°C for 10 min, separated by SDS-PAGE on 10% gels, and transferred to poly vinylidene difluoride (PVDF) membranes (BIO-RAD 162-0177). The membranes were blocked with 5% non-fat dry milk (SIGMA 70166) in TBS-Tween 20 (0,1%) (SIGMA P1379) for 60 min, incubated with primary antibodies overnight at 4 °C, washed three times with TBS-T for 15 min, incubated with the peroxidase-conjugated secondary antibody (1:2000 Amersham Biosciences NA931V) in TBS-T with 5% non-fat dry milk for 60 min, and washed three times with TBS-T for 10 min. Immunoreactive proteins were detected using Pierce ECL Western Blotting Substrate (Thermo Scientific 32106).

Statistical Analysis

The data are represented as means ± SE of independent experiments and statistical significance is calculated by unpaired t-Test. Statistical significance is represented by *P<0,05, **P<0,01, ***P<0,001.

Drug treatment

The cytoskeleton inhibitors were used as indicated in the table below during all the co-culture steps.

Inhibitors	Tested concentration (μM)	Company
**Y-27632**	10 - 20 - 40	BD biosciences (562822)
**Cytochalasin D**	0.1 - 0.5 - 1	SIGMA (C8273)
**Blebbistatin**	10 - 25 - 50	Abcam (ab120425)

Supplementary Table 1. Antibodies used in the article with corresponding working dilution.

Antibodies	Working concentration	Species	Company
**α-Actin**	IF 1:500	Mouse monoclonal	ABCAM (ab8226)
**α-ß-catenin**	IF 1:500	Mouse monoclonal	BD transduction laboratories (610153)
**α-E-cadherin**	WB 1:1000	Mouse monoclonal	BD transduction laboratories (610182)
**α-E-cadherin**	FC 0,5 μg/10^6 cells	Rat IgG1, k	Biolegend (147308)
**α- ß-tubulin**	WB 1:1000	Mouse monoclonal	SIGMA (T0198)
**α-Mac-1**	FC 0,5 μg/10^6 cells	Rat monoclonal IgG2,k	eBioscience (17-0112)
**α-SSEA-1**	Opsonization 0,5 μg/10^6 cells	Mouse IgM, k	Biolegend (125607)

2. Supplemental reference

1. Conti, L. *et al.* Niche-independent symmetrical self-renewal of a mammalian tissue stem cell. *PLoS Biol* **3**, e283 (2005).
2. Ying, Q.L., Nichols, J., Evans, E.P. & Smith, A.G. Changing potency by spontaneous fusion. *Nature* **416**, 545-548 (2002).
3. Welm, B.E., Dijkgraaf, G.J., Bledau, A.S., Welm, A.L. & Werb, Z. Lentiviral transduction of mammary stem cells for analysis of gene function during development and cancer. *Cell Stem Cell* **2**, 90-102 (2008).
4. Kanda, T., Sullivan, K.F. & Wahl, G.M. Histone-GFP fusion protein enables sensitive analysis of chromosome dynamics in living mammalian cells. *Curr Biol* **8**, 377-385 (1998).

3. Supplementary Figure Legends

Supplementary Figure S1. Co-culture of MSCs and ESCs originate heterotypic hybrids *in vitro*.

(**a**) Representative FACS analysis of the cells transduced with human immunodeficiency lentiviral particles carrying HIV-H2B::mRFP or eGFP. (**b**) ESCs-mRFP, MSCs-eGFP or hybrid cells were fixed and stained with DAPI to assess DNA content by FACS.

Supplementary Figure S2. Phagocytosis is not responsible for bi-nucleated cell formation

(**a**) Representative electron microscopy pictures of FACS-sorted $eGFP^+/mRFP^+$ cells derived from 6 hrs co-cultures of ESCs-mRFP and MSCs-eGFP. Left and right images show internalised ESC and heterokaryon, respectively. Blue pseudo-colour represents the nuclei (scale bar 2 μm). (**b**) FACS analysis of Mac-1 staining in ESCs and MSCs shows no expression of the macrophage marker Mac-1. (**c**) ESC opsonisation by SSEA-1 antibody does not enhance $eGFP^+/mRFP^+$ formation. Data are represented as means ± SE (number of independent experiments n=2). (**d**) FACS analysis of AnxV staining in ESCs and MSCs after 6 hrs of co-culture shows no exposure of phosphatidylserine (PS) on the plasma membrane outer leaflet.

Supplementary Figure S3. Heterokaryons but not entotic-derived hybrids expose E-cadherin on plasma membrane

(**a**) Experimental scheme representing the approach to quantify and separate entotic from fused cells based on E-cad expression. (**b**) Western blot analysis showing E-cad expression in ESCs, HIV-H2B::mRFP infected ESCs, MSCs and HIV-H2B-eGFP infected MSCs. Tubulin was used as a loading control. (**c**) Representative FACS analysis of mixed ESCs and MSCs after being 45 min in suspension and 6 hrs in adhesion. (**d**) Imaging flow cytometry gating strategy to identify the $eGFP^+/mRFP^+$ cells and representative images of fused (upper panel) and entotic cells (lower panel).

Supplementary Figure S4. The Rho pathway is essential for fusion and entosis.

(**a-c**) Change of MSC and ESC morphology when treated with increasing dosage of cytochalasin D (**a**), blebbistatin (**b**) and Y-27632 (**c**) are shown in bright field pictures (scale bar 50 μm). (**d**) Quantitative Real Time PCR of ESCs and MSCs infected with

lentiviruses carrying short hairpin control (*shCTR*) or *shRock1.1* (*shR1.1*), *shRock1.2* (*shR1.2*), *shRock2.1* (*shR2.1*), *shRock2.2* (*shR2.2*) as indicated (technical replicates). (**e**) Representative pictures of ESCs and MSCs morphology after transduction with lentiviruses carrying *shCTR* or *shR1.1* or *shR2.1* (scale bar 50 μm).

Supplementary Figure S5. Analysis of cell-to-cell doublet formation.
(**a-b**) Representative FACS gating strategy used to exclude cell-cell aggregates (forward scatter area vs forward scatter height) after cytD, blebblistatin and Y-27632 treatments (**a**) or *shRock1* and *shRock2* co-culture experiments (**b**). (**c**) Quantification of singlet and cell-cell doublets after cytD, blebblistatin and Y-27632 treatments in co-culture experiments. Data are represented as means ± SE (number of indipendent experiment n=9). (**d**) Quantification of singlet and cell-cell doublets in *shRock1* and *shRock2* co-culture experiments. Data are represented as means ± SE (number of indipendent experiment n=5)

4. Supplementary movie legend

Supplementary Movie S1, S2

Time-lapse analysis of FACS-sorted $eGFP^+/mRFP^+$ cells derived from ESC and MSC co-cultures. Movies show heterokaryons characterised by two nuclei into the same cytoplasm that exchange H2B-eGFP or mRFP fusion proteins. DIC, H2B-eGFP and H2B-mRFP images were taken every 5 min (time is shown as hours:minutes).

Supplementary Movies S3, S4

Time-lapse analysis of FACS-sorted $eGFP^+/mRFP^+$ cells derived from ESC and MSC co-cultures. Movies show ESC internalization into MSCs. DIC, H2B-eGFP and H2B-mRFP images were taken every 6 and 3 min for S1 and S2 respectively (time is shown as hours:minutes).

Supplementary Movie S5

Time-lapse analysis of FACS-sorted $eGFP^+/mRFP^+$ cells derived from ESC and MSC co-cultures. Movie shows internalized ESC into MSCs undergoing degradation. White and yellow arrows indicate late stage of degradation characterized by the spreading of entotic vacuole contents to the lysosomal network. DIC, H2B-eGFP and H2B-mRFP images were taken every 1 min (time is shown as hours:minutes).

Supplementary Movie S6

Time-lapse analysis of FACS-sorted $eGFP^+/mRFP^+/E\text{-}cad^-$ cells derived from ESC and MSC co-cultures. Movie shows internalised ESCs into MSCs undergoing cell division. DIC, H2B-eGFP and H2B-mRFP images were taken every 13 min (time is shown as hours:minutes).

Supplementary Movie S7

Time-lapse analysis of FACS-sorted $eGFP^+/mRFP^+/E\text{-}cad^-$ cells derived from ESC and MSC co-cultures. Movie shows internalised ESC into MSCs undergoing cell death. DIC, H2B-eGFP and H2B-mRFP images were taken every 6 min (time is shown as hours:minutes).

Supplementary Movie S8

Time-lapse analysis of FACS-sorted $eGFP^{+}/mRFP^{+}/E\text{-}cad^{-}$ cells derived from ESC and MSC co-cultures. Movie shows internalised ESC escape from MSC cytoplasm. DIC, H2B-eGFP and H2B-mRFP images were taken every 6 min (time is shown as hours:minutes).

Supplementary Movie S9

Time-lapse analysis of FACS-sorted $eGFP^{+}/mRFP^{+}/E\text{-}cad^{+}$ cells derived from ESC and MSC co-cultures. Movie shows heterokaryons undergoing cell death. DIC, H2B-eGFP and H2B-mRFP images were taken every 5 min (time is shown as hours:minutes).

Supplementary Movie S10 and S11

Time-lapse analysis of FACS-sorted $eGFP^{+}/mRFP^{+}/E\text{-}cad^{+}$ cells derived from ESC and MSC co-cultures. Movies show heterokaryon undergoing cell division to generate two daughter synkaryons. DIC, H2B-eGFP and H2B-mRFP images were taken every 5 min (time is shown as hours:minutes).

5. Supplementary Figures

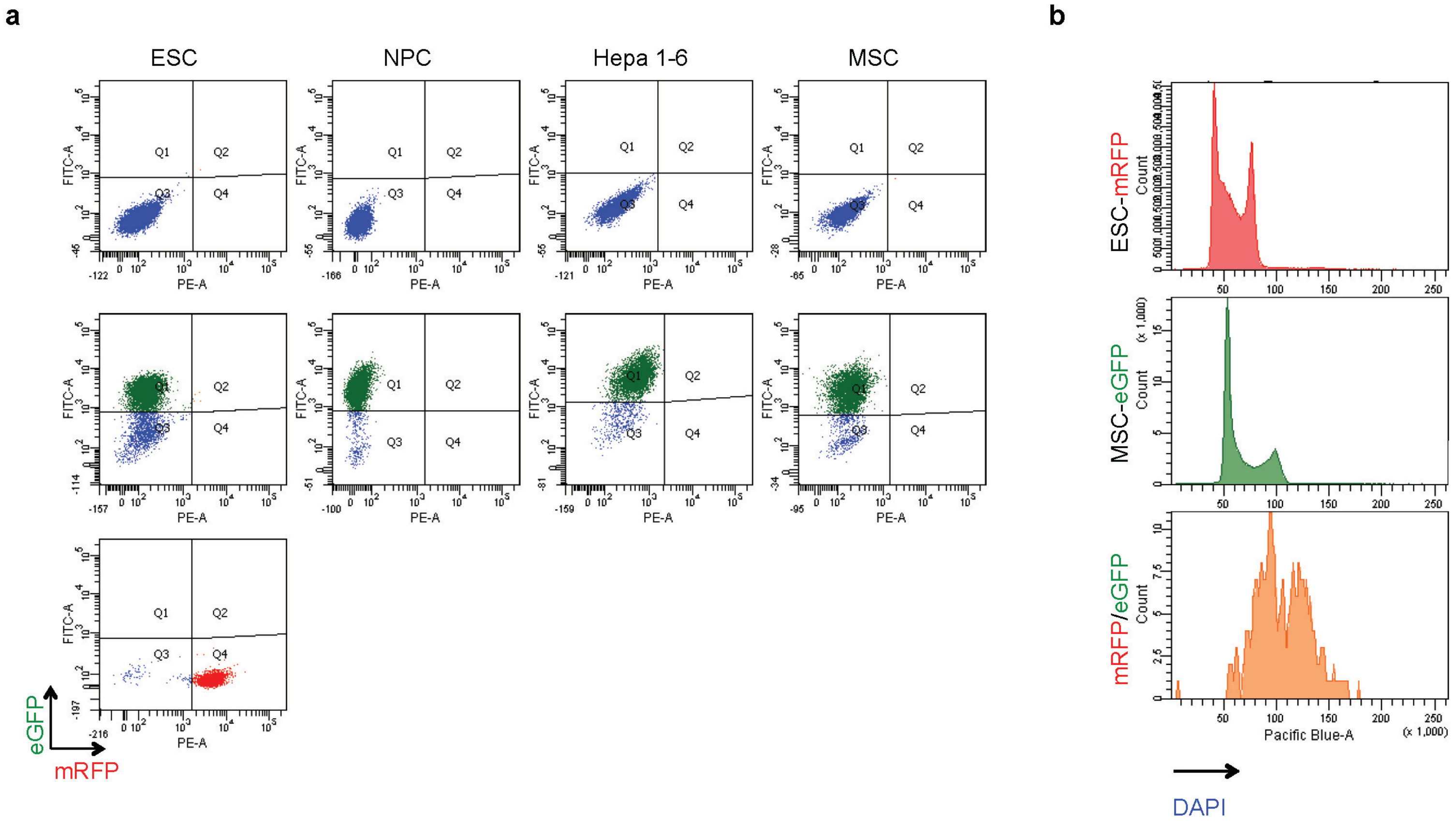
a
b
ESC
NPC
Hepa 1-6
MSC
FITC-A
PE-A
Q1
Q2
Q3
Q4
eGFP
mRFP
ESC-mRFP
MSC-eGFP
mRFP/eGFP
Count
(x 1,000)
Pacific Blue-A
DAPI

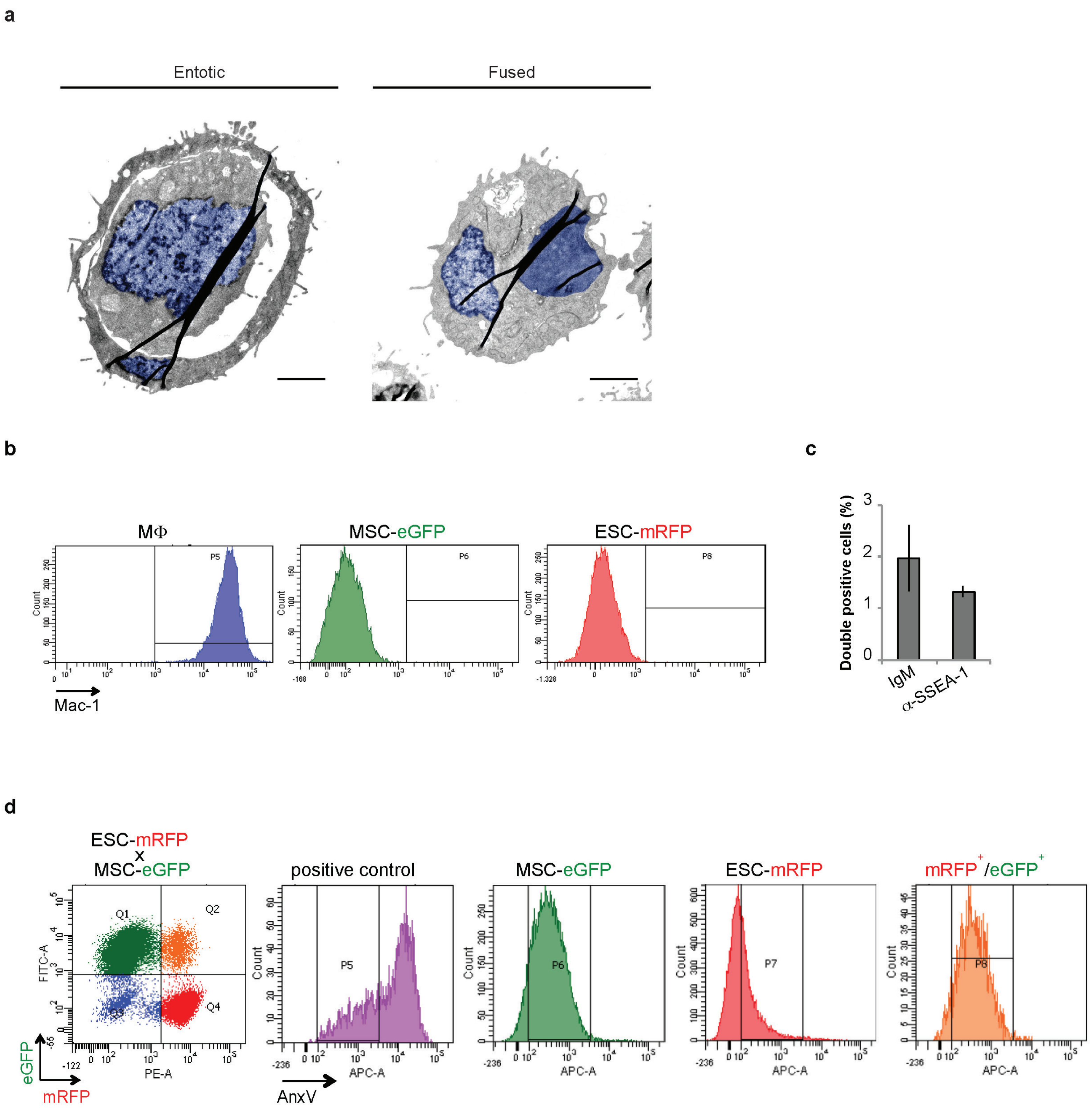
a
Entotic
Fused
b
MΦ
MSC-eGFP
ESC-mRFP
P5
P6
P8
Count
Mac-1
c
Double positive cells (%)
IgM
α-SSEA-1
d
ESC-mRFP
x
MSC-eGFP
positive control
MSC-eGFP
ESC-mRFP
mRFP+/eGFP+
Q1
Q2
Q3
Q4
FITC-A
PE-A
eGFP
mRFP
P5
P6
P7
P8
Count
APC-A
AnxV

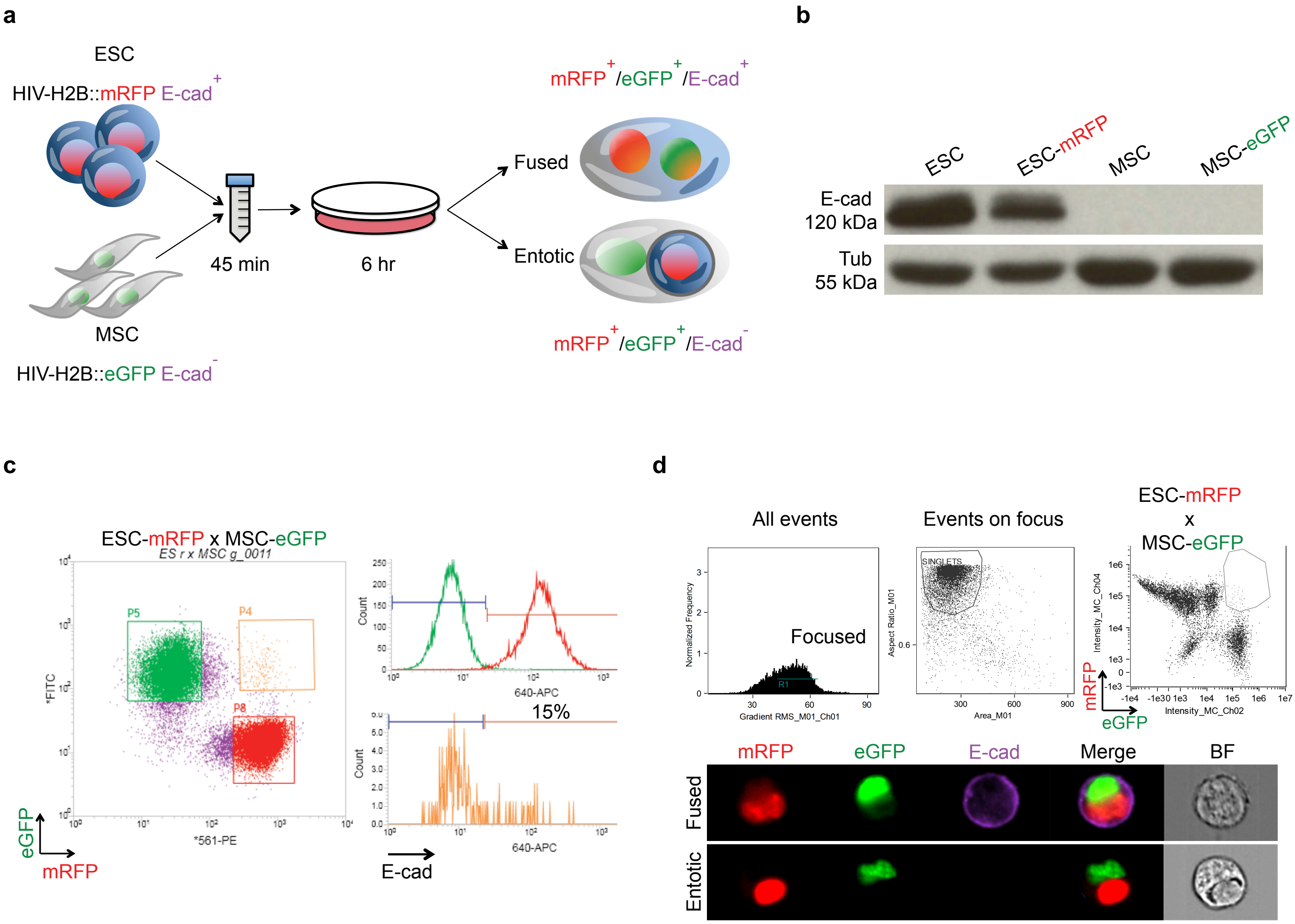

a
ESC
HIV-H2B::mRFP E-cad+
MSC
HIV-H2B::eGFP E-cad-
45 min
6 hr
Fused
Entotic
mRFP+/eGFP+/E-cad+
mRFP+/eGFP+/E-cad-
b
ESC
ESC-mRFP
MSC
MSC-eGFP
E-cad
120 kDa
Tub
55 kDa
c
ESC-mRFP x MSC-eGFP
ES r x MSC g_0011
P5
P4
P8
*FITC
*561-PE
eGFP
mRFP
Count
640-APC
15%
E-cad
d
All events
Events on focus
ESC-mRFP
x
MSC-eGFP
Focused
Normalized Frequency
Gradient RMS_M01_Ch01
SINGLETS
Aspect Ratio_M01
Area_M01
Intensity_MC_Ch04
Intensity_MC_Ch02
mRFP
eGFP
E-cad
Merge
BF
Fused
Entotic

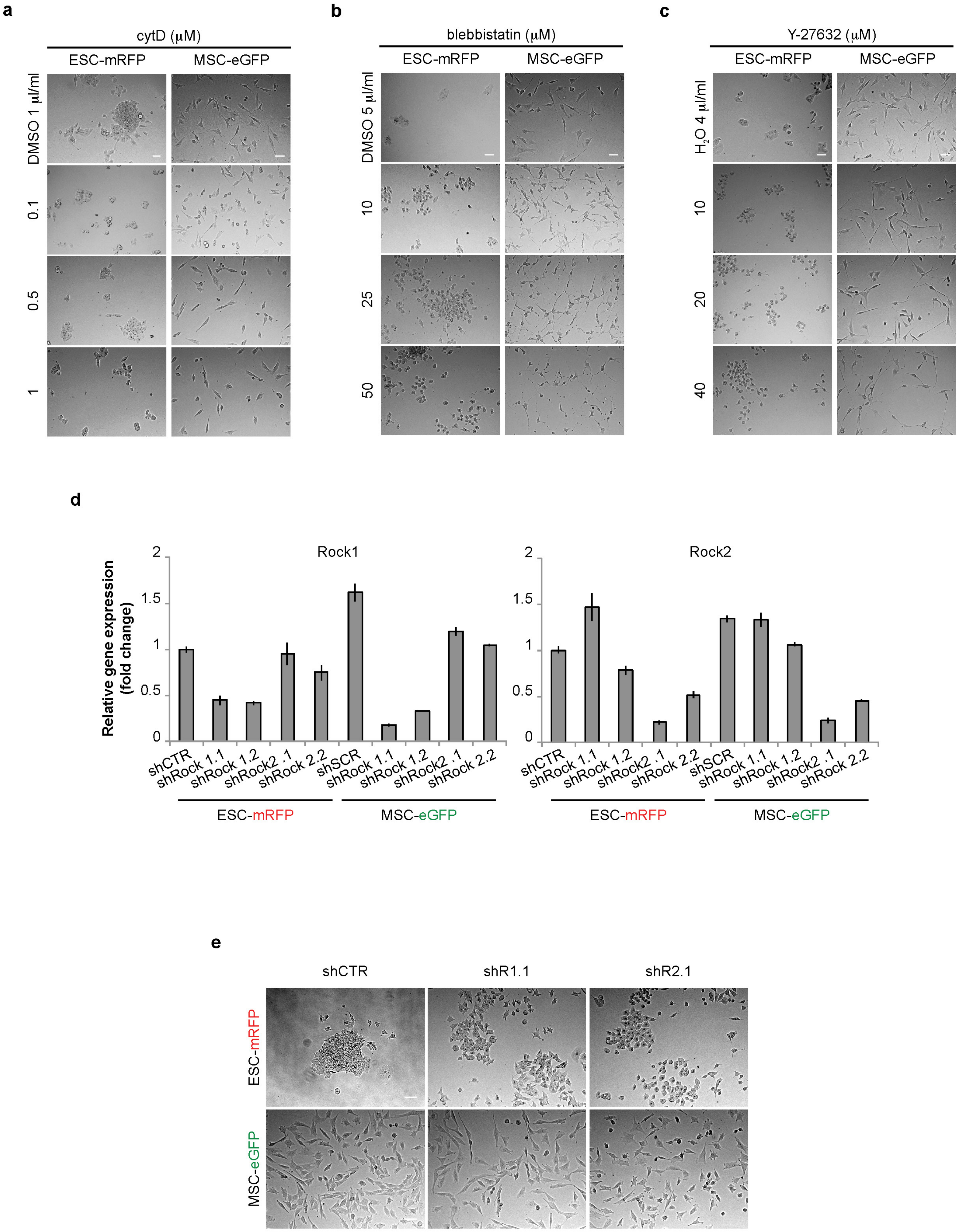
a
cytD (μM)
ESC-mRFP
MSC-eGFP
DMSO 1 μl/ml
0.1
0.5
1
b
blebbistatin (μM)
ESC-mRFP
MSC-eGFP
DMSO 5 μl/ml
10
25
50
c
Y-27632 (μM)
ESC-mRFP
MSC-eGFP
H2O 4 μl/ml
10
20
40
d
Relative gene expression (fold change)
Rock1
Rock2
2
1.5
1
0.5
0
shCTR
shRock 1.1
shRock 1.2
shRock2 .1
shRock 2.2
shSCR
shRock 1.1
shRock 1.2
shRock2 .1
shRock 2.2
ESC-mRFP
MSC-eGFP
e
shCTR
shR1.1
shR2.1
ESC-mRFP
MSC-eGFP

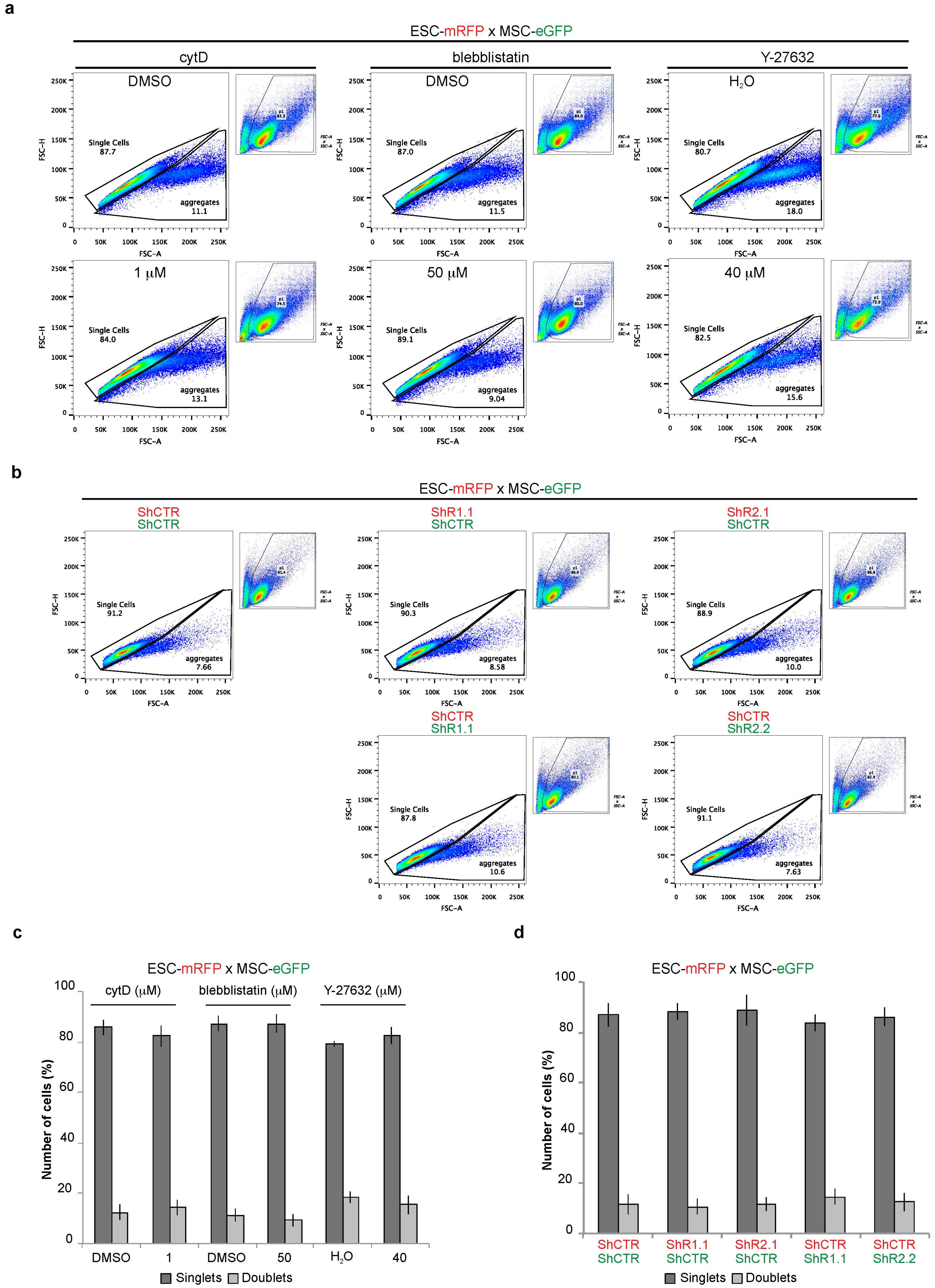
a
ESC-mRFP x MSC-eGFP
cytD
blebblistatin
Y-27632
DMSO
DMSO
H_2O
Single Cells 87.7
aggregates 11.1
Single Cells 87.0
aggregates 11.5
Single Cells 80.7
aggregates 18.0
1 μM
50 μM
40 μM
Single Cells 84.0
aggregates 13.1
Single Cells 89.1
aggregates 9.04
Single Cells 82.5
aggregates 15.6
FSC-A
FSC-H
0
50K
100K
150K
200K
250K
b
ESC-mRFP x MSC-eGFP
ShCTR ShCTR
ShR1.1 ShCTR
ShR2.1 ShCTR
Single Cells 91.2
aggregates 7.66
Single Cells 90.3
aggregates 8.58
Single Cells 88.9
aggregates 10.0
ShCTR ShR1.1
ShCTR ShR2.2
Single Cells 87.8
aggregates 10.6
Single Cells 91.1
aggregates 7.63
c
ESC-mRFP x MSC-eGFP
cytD (μM)
blebblistatin (μM)
Y-27632 (μM)
Number of cells (%)
0
20
40
60
80
100
DMSO
1
DMSO
50
H_2O
40
Singlets
Doublets
d
ESC-mRFP x MSC-eGFP
Number of cells (%)
ShCTR ShCTR
ShR1.1 ShCTR
ShR2.1 ShCTR
ShCTR ShR1.1
ShCTR ShR2.2
Singlets
Doublets
